# The mediating role of feedback-seeking behavior in the relationship between self-efficacy and transition shock of nursing interns: a cross-sectional study

**DOI:** 10.3389/fmed.2025.1635755

**Published:** 2025-10-31

**Authors:** Sai Liang, Mingyu Xue, Naifu Tang, Han Ban, Yanru Qin, Yongyi Chen

**Affiliations:** ^1^Department of Oncology, The First Affiliated Hospital of Zhengzhou University, Zhengzhou, Henan, China; ^2^Department of Emergency, The First Affiliated Hospital of Zhengzhou University, Zhengzhou, Henan, China; ^3^Department of Nursing, The Affiliated Tumor Hospital of Xiangya School of Medicine, Central South University, Changsha, Hunan, China

**Keywords:** self-efficacy, nursing interns, feedback-seeking behavior, transition shock, cross-sectional study

## Abstract

**Background:**

Transition shock commonly occurs as nursing interns move from student roles to professional practice, leading to confusion, uncertainty, and a lack of clarity in their psychological, physiological, knowledge and skills development.

**Aim:**

This study aims to investigate the status of transition shock among Chinese nursing interns and explore the mediating role of feedback-seeking behavior in the relationship between self-efficacy and transition shock.

**Methods:**

This is a cross-sectional survey study following the STROBE guidelines. A convenience sample of 450 nursing interns were surveyed from February to March 2025. Participants completed a questionnaire that included socio-demographic information, Transition Shock Scale, General Self-Efficacy Scale (GSES), and Feedback-Seeking Behavior Scale. The Bootstrap method was applied to assess the mediating effects.

**Results:**

The mean ± SD for transition shock among nursing interns was 59.28 ± 4.53. Factors influencing transition shock included gender, reasons for choosing nursing, physical health status, enjoyment of the nursing major, class leadership roles, only-child status, and parental education levels. Self-efficacy (*r* = −0.651, *p* < 0.001) and feedback-seeking behavior (*r* = −0.711, *p* < 0.001) were negatively correlated with transition shock. Feedback-seeking behavior was found to mediate the relationship between self-efficacy and transition shock (indirect effect = −0.585, 95% CI: −0.766 to −0.389), accounting for 66.1% of the total effect.

**Conclusion:**

Feedback-seeking behavior is the mediating variable between self-efficacy and transition shock of nursing interns. These insights provide evidence-based strategies for targeted interventions aimed at alleviating transition shock among nursing interns.

## 1 Introduction

According to the report of the World Health Organization (WHO), the total number of nurses currently is far from meeting the demand for health care services. The WHO Global Strategy on Human Resources for Health estimated that by 2030 there would be a global shortage of 7.6 million nurses and midwives in countries with a density below a benchmark of 4.45 physicians (1). In China, the turnover rate of nurses ranges from 0.64 to 12.71%, which may reflect regional and institutional differences in healthcare systems and working conditions (2). Nursing interns are an important reserve force for new nurses. The purpose of the nursing internship is to provide necessary professional skills for nursing interns and ensure that they can provide high-quality nursing and healthcare services in the future, thus smoothly transitioning into registered nurses (3). However, as nursing interns transition from student roles to practicing nurses, they frequently encounter confusion, doubts, and unclear role definitions. This transition shock is influenced by their limited knowledge, evolving responsibilities, and interpersonal relationships. It impacts them across multiple dimensions, including psychological, physiological, and developmental aspects related to knowledge, skills, and social and cultural integration. Transition shock primarily manifests in four key areas: physical well-being, psychological adaptation, knowledge and skills acquisition, and social and cultural development (4). Previous research has indicated that nursing interns experiencing transition shock frequently encounter issues such as tension and anxiety. A failure to navigate this transition smoothly not only poses safety risks during their internship but also adversely affects their personal growth and professional development (5). Many nursing students tend to quit the nursing profession after their clinical practice, which in turn affects the stability of the nursing workforce (6, 7). The clinical internship period represents a critical phase in the transformation of nursing students into specialized nurses, making it an opportune time to address transition shock. Consequently, focusing on this stage to investigate the impacts of the transition and its influencing factors is of significant importance.

Self-efficacy is a core concept of Bandura’s (8) social cognitive theory, referring to an individual’s confidence in their ability to successfully complete a specific task. It is one of the important determinants of behavior. The self-efficacy of nursing interns is reflected in their confidence levels regarding clinical skills, decision-making abilities, and the capacity to handle unexpected situations. Individuals with low self-efficacy may avoid feedback due to the fear of being exposed for their inadequacies (9). Research shows that high self-efficacy can enhance the resilience of nursing interns when facing challenges, promote active learning and adaptive behaviors. Preliminary studies have found that higher self-efficacy is associated with lower transition shock in new nurses (5, 10). However, evidence is limited for nursing interns regarding the relationship between self-efficacy and transition shock, considering that the intern stage is critical for future career orientation.

Feedback-seeking behavior refers to an individual’s proactive behavior of obtaining valuable information through active observation or inquiry of their leaders, mentors, colleagues, etc., in order to adapt to the needs of organizational and personal development (11). Proactive feedback-seeking behaviors enable new employees to adapt to the requirements of their careers as soon as possible, enhance their work enthusiasm and improve their work efficiency (12). This kind of behavior is regarded as a positive learning strategy, which can help individuals identify their own shortcomings, adjust their behavioral patterns and enhance their professional capabilities (13, 14). In the clinical setting, the behavior of feedback-seeking has been proven to be closely related to higher levels of occupational adaptability (15). A cross-sectional study has found that the feedback-seeking behavior of nursing intern students can reduce the level of transition shock, and meanwhile, it has a positive impact on their attitudes towards future nursing careers (16).

**Figure 1:**
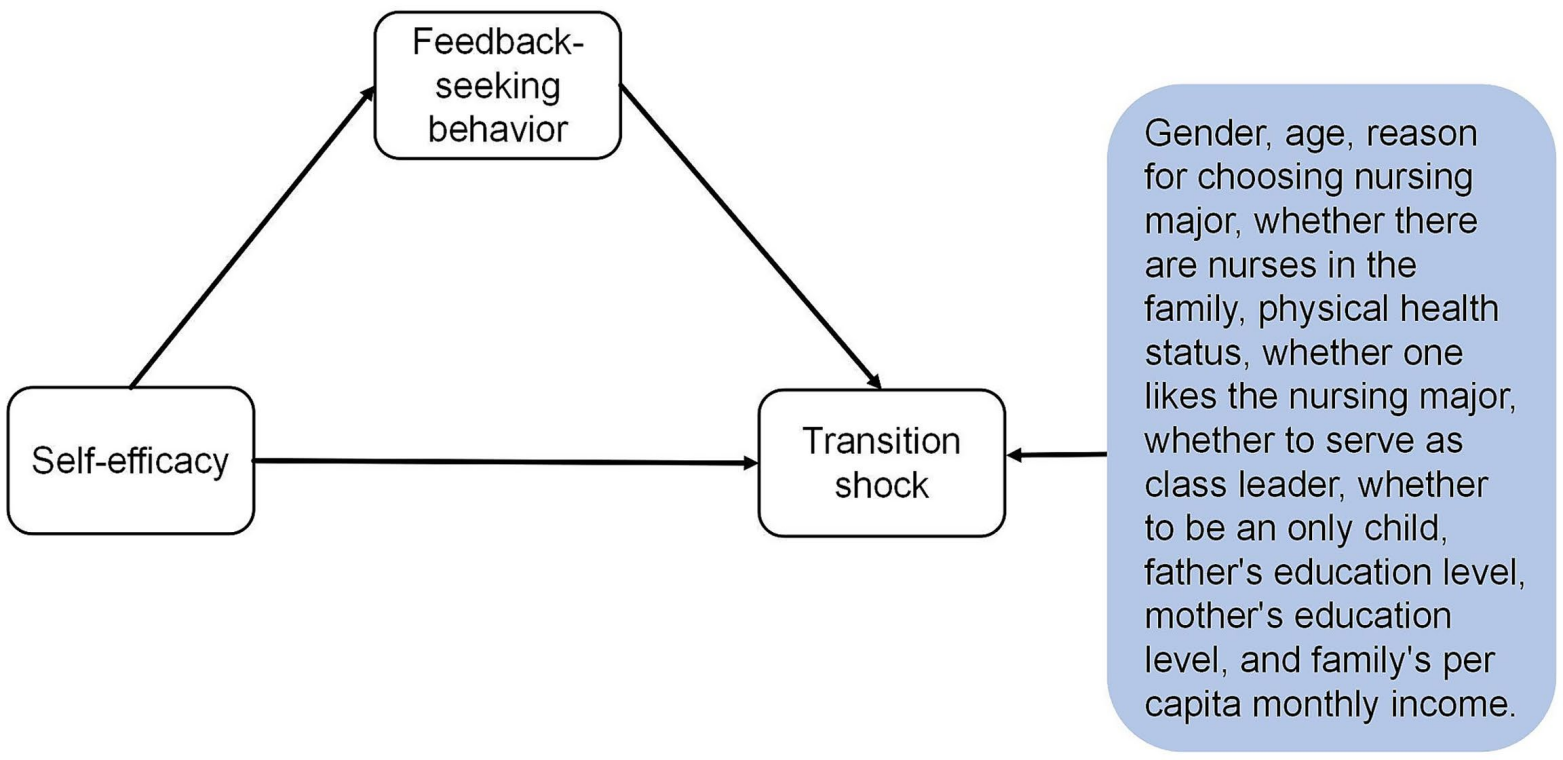
Hypothetical relationships between self-efficacy, feedback-seeking behavior and transition shock.

In summary, the factors influencing transition shock are multi-faceted. Most existing research has primarily examined the direct relationships between self-efficacy and transition shock, as well as feedback-seeking behavior and transition shock, with a predominant focus on newly graduated nurses. However, the interplay between self-efficacy and feedback-seeking behavior among nursing interns, and whether feedback-seeking behavior mediates the relationship between self-efficacy and transition shock, warrant further investigation. Therefore, this study specifically targeted nursing interns with the following objectives: (1) to assess the current prevalence of transition shock among nursing interns, and (2) to explore the mediating role of feedback-seeking behavior in the relationship between self-efficacy and transition shock. We hypothesize that: (1) transition shock is negatively correlated with both self-efficacy and feedback-seeking behaviors; and (2) feedback-seeking behavior mediates the relationship between self-efficacy and transition shock among nursing interns (see Figure 1).

## 2 Methods

### 2.1 Study design

From February 18th to March 19th, 2025, a cross-sectional study was conducted in China, and this study was reported in accordance with the STROBE guideline (17).

### 2.2 Setting and sample

The target population consists of clinical nursing interns from diverse regions across China. Initially, Henan Province, located in central China, was selected as the primary sample. This province comprises 17 prefectures, from which one prefecture was randomly chosen using a random sampling method. Within this prefecture, nursing interns from a tertiary grade A hospital were subsequently selected at random. Following this initial selection, a random sample was drawn, and the snowball sampling technique was employed, allowing participants to recommend additional individuals who met the inclusion criteria for the study. Inclusion criteria for this study comprise nursing interns who have completed their professional coursework, are currently engaged in clinical practice, have not yet obtained their nursing qualification certificates, are willing to participate in the research, and demonstrate good compliance. Exclusion criteria include individuals who are already employed as nurses, those enrolled in training programs, visiting scholars, and participants unable to continue throughout the duration of the study. An invalid questionnaire is defined as one in which all items are answered with the same option.

Using Kendall’s counting method for sample size estimation (18), the required sample size for this study should be between 5 to 10 times the number of variable dimensions. This study encompasses 31 variables: the general information questionnaire (11 dimensions), the general self-efficacy scale (10 dimensions), the feedback-seeking behavior scale (4 dimensions), and the transition shock evaluation scale (6 dimensions). Consequently, the sample size is determined to be between 155 and 310 cases. To account for an estimated 20% of invalid questionnaires, the adjusted sample size should range from 194 to 388 cases (calculated as 155/0.8 to 310/0.8 cases).

### 2.3 Measures

The social demographic characteristics of the sample include 11 variables: gender, age, reason for choosing nursing major, whether there are nurses in the family, physical health status, whether one likes the nursing major, whether to serve as class leader, whether to be an only child, father’s education level, mother’s education level, and family’s per capita monthly income.

The General Self-Efficacy Scale (GSES) compiled by Schwarzer et al., translated and revised by Zhang et al. (19) was used to assess the self-efficacy of nursing interns. This scale is used to assess students’ general self-efficacy, which is a single dimension and consists of 10 items. A scoring system ranging from 1 (completely incorrect) to 4 (completely correct) is adopted. The total score ranges from 10 to 40. The higher the score, the higher the individual’s level of self-efficacy. The Cronbach’s alpha coefficient of this scale in this study is 0.95, indicating good internal consistency.

The Feedback-seeking Behavior Scale was used to assess the feedback-seeking behavior of nursing interns. This scale was developed by Callister et al. (11), and was translated and revised by Gong et al. (14). Lian et al. (20) applied this scale to measure the feedback-seeking behavior of intern nursing students, and the Cronbach’s alpha coefficient of the scale was 0.89, including 4 dimensions and 11 items: observation-based feedback-seeking from leaders (2 items), inquiry-based feedback-seeking from leaders (2 items), observation-based feedback-seeking from colleagues (3 items), and inquiry-based feedback-seeking from colleagues (4 items). To adapt the scale for nursing interns, we conducted a pilot test with 30 students to assess clarity and relevance. Minor wording adjustments were made based on feedback: changing “leaders” to “mentors” and “colleagues” to “students”. This scale uses the 7-point Likert scoring method, scores ranging from “strongly disagree” to “strongly agree” are assigned with values from 1 to 7, respectively. The total score ranges from 11 to 77, with higher scores indicating better feedback-seeking behavior. The Cronbach’s alpha coefficient of this scale in our study is 0.98.

The Transition Shock Scale was used to evaluate the transition shock levels of nursing interns. This scale was developed by Kim et al. (21), and was introduced to Chinese by Huang et al. in 2021 for cultural adaptation (22). It includes six dimensions and 18 items, namely theoretical and practical conflicts (3 items), excessive workload during internship (3 items), lack of social support (2 items), tense interpersonal relationships (3 items), confusion regarding nursing professional values (5 items), and incompatibility between clinical internship and personal life (2 items). This scale uses the 4-point Likert scoring method, ranging from 1 (strongly disagree) to 4 (strongly agree). The score range is from 18 to 72. The higher the score, the greater the impact of the nursing students’ transition. The Cronbach’s alpha coefficient of this scale in this study is 0.79.

### 2.4 Data collection

Online questionnaire surveys were conducted using the Questionnaire Star platform.^1^ The introduction to the questionnaire included a standardized set of instructions detailing the survey’s purpose, confidentiality principles, and guidelines for completing the questionnaire. Following the participants’ consent, the questionnaires were filled out anonymously and independently.

Initially, the three authors of this study served as data collection investigators. They underwent standardized training to ensure a comprehensive understanding of the research objectives and accurate interpretation of the questionnaire precautions. The investigator then contacted the head instructor at the hospital to obtain verbal consent, subsequently adding the instructor’s WeChat (a social media platform in China) account to join the working group.

Investigators randomly selected participants who met the inclusion criteria from this group and communicated the research content, as well as data privacy and protection measures. Participants were invited to scan a QR code to access the online questionnaire. Those who had already participated were allowed to recommend additional candidates who met the inclusion criteria. To prevent information discrepancies, all questions were designated as mandatory, and responses from the same IP address or mobile phone number were limited to one submission.

As of March 19, 2025, a total of 451 nursing interns had completed the questionnaire. After excluding one invalid response (where all answers were identical), 450 valid questionnaires were retained, meeting the required sample size.

### 2.5 Data analysis

Data analysis was performed using R software (version 4.4.1) for statistical evaluation. The Shapiro–Wilk test was employed to assess the normality of continuous data. For normally distributed data, means (M) and standard deviations (SD) were used for description. In cases where the data did not follow a normal distribution, medians and interquartile ranges (IQR) were reported. Categorical variables were summarized using frequencies and percentages. Independent sample *t*-tests and one-way analysis of variance (ANOVA) were utilized to evaluate differences in transition shock for those with different socio-demographic characteristics. Pearson correlation analysis was conducted to examine the relationships among transition shock, self-efficacy, and feedback-seeking behavior. Additionally, multiple linear regression analysis was performed to identify the associations between self-efficacy, feedback-seeking behavior and transition shock after adjusted for confounding covariates. Finally, the R package “mediation” was then used to test the mediation model, employing the Bootstrap method to assess the mediating effect of feedback-seeking behavior on the relationship between self-efficacy and transition shock. A total of 1,000 bootstrap samples were generated to calculate the 95% confidence interval for the indirect effect. A two-sided *p*-value of < 0.05 was considered statistically significant.

### 2.6 Patient and public involvement

There was no patient or public involvement in the study.

### 2.7 Ethical considerations and consent to participate

This study has been approved by the Ethics Committee of the First Affiliated Hospital of Zhengzhou University (Zhengzhou, Henan Province, China) (Ethical Approval Number: 2022-KY-540). All methods were carried out in accordance with relevant guidelines and regulations. Participants had given informed consent to participate in the study before they joined the research.

## 3 Results

### 3.1 Sample characteristics

A total of 451 clinical nursing interns participated in the survey. The distribution of participants by province was as follows: 166 from Hebei (36.81%), 63 from Henan (13.97%), 63 from Jiangsu (13.97%), 32 from Zhejiang (7.10%), 16 from Jiangxi (3.55%), 15 from Shandong (3.33%), 14 from Guangdong (3.10%), 13 from Anhui (2.88%), 12 from Hubei (2.66%), 10 from Sichuan (2.22%), 9 from Fujian (2.00%), 8 from Liaoning (1.77%), 5 from Guangxi (1.11%), 5 from Beijing (1.11%), 5 from Hunan (1.11%), 5 from Shanxi (1.11%), 2 from Xinjiang (0.44%), 2 from Gansu (0.44%), 1 from Qinghai (0.22%), 1 from Jilin (0.22%), 1 from Shanghai (0.22%), and 1 from Tianjin (0.22%). After excluding one invalid questionnaire, 450 valid responses remained, yielding an effective response rate of 99.8%.

Among the participants, 163 were male (36.2%) and 287 were female (63.8%). The average age of the respondents was 23.13 ± 3.19 years, with an average internship duration of 9.20 ± 0.79 months. The mean self-efficacy score was 20.67 ± 7.25, the mean feedback-seeking behavior score was 35.98 ± 21.79, and the mean transition shock score was 59.28 ± 4.53. Descriptive statistics for the 450 clinical nursing interns are summarized in Table 1.

**Table 1:** Participants’ demographics and differences in transition shock (*N* = 450).

Characteristics	Categories	No. (%)	Transition shock		
			Mean ±SD	t/F	*p*
Gender	Male	163 (36.2)	57.7 ± 10.9	3.7	<0.001
	Female	287 (63.8)	53.6 ± 11.2		
Reason for choosing nursing major	Self-interest	118 (26.2)	56.9 ± 11.8	6.4	0.002
	Recommended by family/friends	200 (44.4)	53.0 ± 11.5		
	Others	132 (29.3)	56.6 ± 9.8		
Whether there are nurses in the family	Yes	181 (40.2)	56.2 ± 10.8	1.7	0.085
	No	269 (59.8)	54.4 ± 11.5		
Physical health status	Health	243 (54.0)	51.8 ± 12.1	25.4	<0.001
	Normal	158 (35.1)	58.7 ± 8.4		
	Ill-health	49 (10.9)	59.9 ± 9.0		
Whether one likes the nursing major	Like	174 (38.7)	56.4 ± 11.6	21.9	<0.001
	Normal	167 (37.1)	51.0 ± 11.1		
	Dislike	109 (24.2)	59.3 ± 8.5		
Whether to serve as class leader	Yes	167 (37.1)	57.6 ± 10.8	3.8	<0.001
	No	283 (62.9)	53.6 ± 11.2		
Whether to be an only child	Yes	143 (29.8)	58.8 ± 9.1	5.2	<0.001
	No	316 (70.2)	53.5 ± 11.7		
Father’s education level	Junior high school and below	186 (41.3)	53.7 ± 12.1	3.7	0.025
	High school or technical secondary school	133 (29.6)	54.9 ± 11.1		
	College or above	131 (29.1)	57.2 ± 9.7		
Mother’s education level	Junior high school and below	212 (47.1)	53.2 ± 11.5	7.7	<0.001
	High school or technical secondary school	130 (28.9)	55.6 ± 11.1		
	College or above	108 (24.0)	58.2 ± 10.1		
Family’s per capita monthly income	Less than 3,000 yuan	120 (26.7)	55.7 ± 11.4	2.1	0.123
	3,000 ~ 5,000 yuan	188 (41.8)	53.8 ± 11.3		
	More than 5,000 yuan	142 (31.6)	56.3 ± 10.9		

### 3.2 Univariate analysis of the impact of different demographic characteristics on the transition shock of nursing interns

The results of this study showed that the transition shock score of males is higher than that of female (57.7 ± 10.9 vs. 53.6 ± 11.2, *p* < 0.001), and the transition shock score of those who are class leaders was higher than that of those who are not (57.6 ± 10.8 vs. 53.6 ± 11.2, *p* < 0.001). The reason for choosing the nursing major (*p* = 0.002), physical health status (*p* < 0.001), whether one likes the nursing major (*p* < 0.001), father’s education level (*p* = 0.025), and mother’s education level (*p* < 0.001) may affect the transition shock score of nursing interns (see Table 1).

### 3.3 Correlation analysis between self-efficacy, feedback-seeking behavior and transition shock in nursing interns

The lower levels of self-efficacy (*r* = −0.651, *p* < 0.001) and feedback-seeking behavior (*r* = −0.711, *p* < 0.001) were associated with higher levels of transition shock (see Table 2).

**Table 2:** Pearson correlation analysis.

Variables	Self-efficacy	Feedback-seeking behavior	Transition shock
Self-efficacy	1		
Feedback-seeking behavior	0.831	1	
Transition shock	−0.651	−0.711	1

All *p* < 0.001.

### 3.4 Mediation effect of feedback-seeking behavior on self-efficacy and transition shock

**Figure 2:**
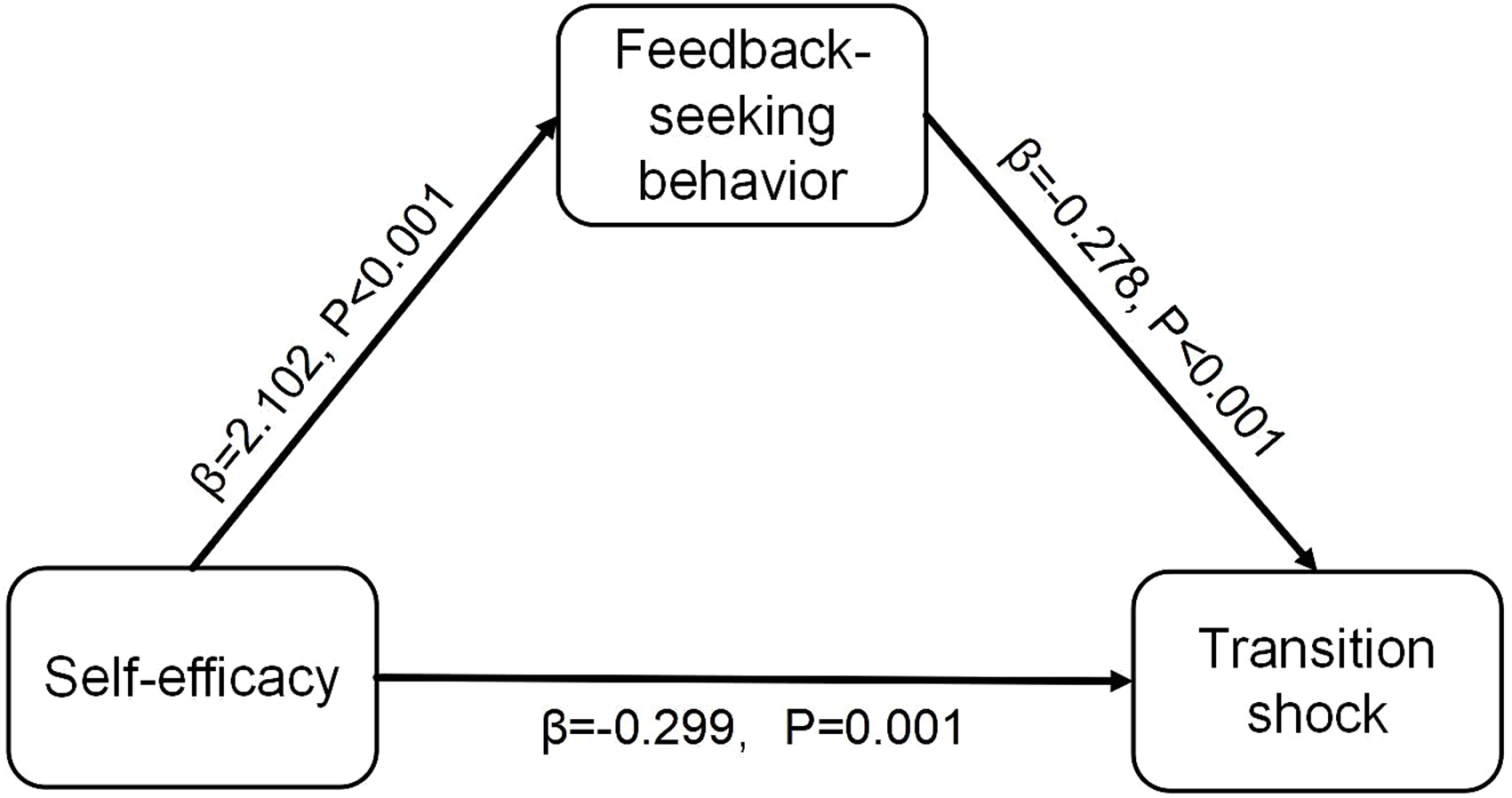
The mediating effect of feedback-seeking behavior in the association between self-efficacy and transition shock.

Tables 3, 4 present the mediating effect of feedback-seeking behavior on the relationship between self-efficacy and transition shock, after adjusting for covariates. The model results are illustrated in Figure 2. Overall, self-efficacy has a significant negative impact on transition shock (*β* = −0.884, *p* < 0.001). Specifically, self-efficacy demonstrates a significant direct effect on transition shock (*β* = −0.299, *p* = 0.001), indicating that higher self-efficacy scores correlate with lower transition shock scores. Furthermore, self-efficacy positively influences feedback-seeking behavior (*β* = 2.102, *p* < 0.001), while feedback-seeking behavior exerts a negative influence on transition shock (*β* = −0.278, *p* < 0.001). Notably, feedback-seeking behavior partially mediates the relationship between self-efficacy and transition shock, with an indirect effect of −0.585 (95% CI: −0.766 to −0.389, *p* < 0.001), accounting for 66.1% of the total effect.

**Table 3:** Multiple linear regression analysis.

Models	*β*	SE	*t*	*p*
Model 1 (outcome: transition shock)				
Self-efficacy	−0.884	0.063	−13.882	<0.001
Model 2 (outcome: feedback-seeking behavior)				
Self-efficacy	2.102	0.083	25.293	<0.001
Model 3 (outcome: transition shock)				
Self-efficacy	−0.299	0.093	−3.206	0.001
Feedback-seeking behavior	−0.278	0.034	−8.115	<0.001

**Table 4:** The results of the mediation effect analysis.

Effects	*β*	95%CI lower	95%CI upper	*p*
Indirect effect	−0.585	−0.766	−0.389	<0.001
Direct effect	−0.298	−0.629	0.005	0.046
Total effect	−0.884	−1.085	−0.679	<0.001
Mediated proportion	0.661	0.401	0.993	<0.001

## 4 Discussion

This study firstly investigated the transition shock status of Chinese clinical nursing interns and examined the mediating role of feedback-seeking behavior in the association between self-efficacy and transition shock.

Of note, all the nursing interns have experienced varying degrees of transition shock. It may be related to factors that nursing students encounter in clinical practice, such as the gap between theory and practice, fear of making mistakes, complexity of communicating with patients, lack of experience in handling emergencies, lack of clinical skills, and inability to cope with patient deaths (23). However, the average score of the transition shock experienced by nursing interns was relatively higher than the cross-sectional study by Tang et al., which was conducted on 564 nursing interns from four tertiary hospitals in the central and southern part of China (7). However, this is lower than the research result of Kim et al. (24) from South Korea. This difference might be related to the sources of the research subjects, the training methods of the nursing students, the healthcare environment, as well as the potential cultural backgrounds between different countries/regions.

Furthermore, the findings of this study reveal that gender, reason for choosing nursing major, physical health status, whether one likes the nursing major, whether to serve as class leader, and whether to be an only child, as well as parents’ education level, are all factors influencing the transition shock of nursing interns.

The results of this study show that the transition shock scores of male clinical nursing interns are higher than those of female ones. The possible reason is that there are differences in the work commitment of nursing interns of different genders during the transition process (25). Studies have shown that the total score of work engagement of female nursing interns is generally higher than that of male ones. This might be related to the higher professional identity of women in the nursing field. However, male nursing interns, lacking clear career planning for the future nursing industry, tend to show lower work engagement during the internship process, thereby demonstrating higher transition shock (26). This is contrary to the findings of Zhao et al. (27). The possible reason is that the proportion of male participants in this study sample is higher than that in Zhao et al.’s study (36.2% vs. 17.4%), and the geographical origin of this study sample is more extensive.

The attitude of nursing interns towards the nursing profession has an independent impact on the level of their transition shock. That is, whether they like the nursing profession has an influence on the level of their transition shock. The reason for choosing the nursing major are also factors influencing the transition shock of nursing interns. This is consistent with the result of Tang et al. which holds that the first choice is related to the transition shock experienced by nursing students (7). The results are consistent with those of Pan et al., who hold the view that different reasons for filling out application forms also affect the outcome of the transition shock of nursing interns (28). The nursing interns who actively chose the nursing major experienced less transition shock. The possible reason is that the environment that has a positive impact on future career choices enables nursing students to adapt to the clinical environment quickly (29). The Self-Consistency Theory holds that an individual’s behavior is guided by the decisions they make, and their decisions are constrained by internal characteristics and external environment. When the external environment aligns with the individual’s internal characteristics, the individual will regard work as a form of self-expression, thereby generating a positive attitude (30).

Moreover, the transition shock experienced by only children is greater than that of non-only children, which is different from the research result of Yao et al. (31). The transition shock endured by students who are class leaders is greater than that of those who are not. The research conducted by Tang et al. (7) suggests that nurses with experience in student leadership can adapt to clinical practice quickly and experience less transition shock, which is inconsistent with the findings of this study. The possible reason is that in the post-pandemic era, nursing students’ perception of the nursing profession has changed (32). Simultaneously, influenced by the new round of medical reform in China and the current economic situation, nursing interns have some concerns about the employment prospects of their major. The parents’ education level is related to the transition shock experienced by nursing students. The possible reason is that the education level of parents varies, which leads to different parenting styles adopted by them towards nursing students, thereby influencing their perception of the profession (32). The physical health condition also has an impact on the transition shock level of nursing students. Studies have shown that healthy behaviors and/or states are conducive to the adaptation and career choice of fresh graduates as nurses (33), which is in line with our findings.

The results of this study also indicate that self-efficacy has a negative impact on the transition shock. The higher the self-efficacy of the nursing interns is, the lower the transition shock they experience. Tong et al. (5) conducted a study on the relationship between the transition shock and self-efficacy of newly graduated nurses. The study confirmed that the transition shock was negatively correlated with self-efficacy, and self-efficacy had a predictive effect on the transition shock experienced by newly graduated nurses. Specifically, the lower the self-efficacy of newly graduated nurses, the stronger the sense of transition shock they would experience. It indicates that nursing interns, like newly graduated nurses, have all experienced varying degrees of transition shock. The possible reason is that nursing interns with high self-efficacy can help improve their self-assessment ability and facilitate the establishment of their growth mindset (23). This further leads to the reduction of the impact of its theory on practice, the interaction between body and mind, and interpersonal relationships. This indicates that nursing managers should pay attention to the self-efficacy level of nursing interns, communicate with them, promptly and effectively address their confusion, provide guidance and support, and instill in them the confidence to confront and solve clinical problems. Therefore, they can actively face the impact of the transition and thereby reduce the level of transition shock.

Furthermore, the feedback-seeking behavior of nursing interns was negatively correlated with the transition shock, indicating that nursing students with higher feedback-seeking behavior showed lower levels of transition shock. Feedback-seeking behavior promotes individual’s autonomous regulation (13). The nursing students with stronger feedback-seeking behavior are better at regulating themselves in their study and life, and they can better solve current problems. They can continuously gain satisfaction from their studies and work as well as good relationships with their classmates, thereby promoting the development of their caring abilities (34). Thereby, it can autonomously adjust and improve itself, overcome the difficulties in clinical practice, and reduce the impact of transformation. This is consistent with the previous research results (16). This suggests that during the clinical internship of nursing students, educators should appropriately provide feedback exchange plans between teachers and students as well as among nursing students themselves. By adopting more diversified teaching models, they can stimulate the feedback-seeking behavior of nursing students. For instance, reflective diaries (35) and role-playing (36) can be employed to promote the feedback-seeking behavior of nursing students and reduce the shock of transition.

On the other hand, the feedback-seeking behavior has a mediating effect between self-efficacy and transition shock. The mediating effect accounts for 66.1% of the total effect of self-efficacy on transition shock. That is, self-efficacy not only affects the transition shock of nursing students, but also indirectly influences their transition shock through feedback-seeking behavior.

Nursing students use feedback-seeking behavior to explore the surrounding environment, express and evaluate it, which is a kind of positive initiative (37). Nursing students with strong feedback-seeking behavior ability are more capable of self-regulation, have stronger self-efficacy, and are good at strengthening communication with clinical nursing seniors, adapting to the transformation from theory to practice and quickly solving problems encountered (26). Ultimately reduce the impact of transformation. The finding that feedback-seeking behavior mediated 66.1% of the total effect highlights its critical role in mitigating transition shock. These findings suggest that nursing educators should integrate structured feedback mechanisms—such as reflective diaries, peer debriefing, and mentorship programs—into clinical training. By fostering an environment that encourages proactive feedback-seeking, educators can help interns build self-efficacy and reduce transition shock. The substantial mediation effect (66.1%) underscores the importance of targeting feedback-seeking behavior as a key intervention strategy.

Our study has shed insights for nursing education practice. Nursing educators can enhance nursing interns’ feedback-seeking behavior by improving their self-efficacy: they can provide clinical practice opportunities, implement personalized teaching based on students’ aptitudes, and help students accumulate direct successful experiences. Additionally, internship experience exchange sessions can be organized to increase indirect experiences through role modeling. Educators should also provide affirmation and encouragement to students, thereby boosting their self-efficacy. Furthermore, understanding the actual needs and mental health conditions of students and establishing a comprehensive support system can help mitigate negative emotional experiences among nursing interns. These strategies collectively aim to strengthen the self-efficacy of clinical nursing interns, promote feedback-seeking behavior, and minimize the impact of transformation challenges. Ultimately, enable nursing interns to complete clinical practice with minimal transitional impact and smoothly transition into registered nurses.

## 5 Strengths and limitations

Overall, this study provides a comprehensive understanding of the transition shock of clinical nursing interns in China and its associated factors, especially the interplay between self-efficacy, feedback-seeking behavior and transition shock. The results of this study provide a basis for hospital nursing teaching to reduce the transition shock of nursing students and to develop more effective intervention measures. However, the study has several limitations. First, this study is a cross-sectional study, so limited causal inference can be drawn between variables, and further longitudinal or mixed-method studies would be necessary to verify our findings in the future. Second, although quality control was conducted before the questionnaire was issued, all assessments were based on self-reported results, and subjects may have some recall bias.

## 6 Conclusion

This study investigated the transition shock of clinical nursing interns and examined the mediating mechanism of feedback-seeking behavior on self-efficacy and transition shock. Specifically, our research shows that most of the clinical nursing students surveyed experienced a transition shock during their clinical placement in the hospital. Gender, reason for choosing nursing major, physical health status, whether one likes the nursing major, whether to serve as class leader, whether to be an only child, and parents’ education level are all influencing factors of transition shock. Of note, higher levels of self-efficacy may not only directly affect the transition shock of clinical nursing interns, but also indirectly affect the transition shock of nursing interns through feedback-seeking behavior. These findings highlight that the transition shock of clinical nursing interns remains a serious problem. Therefore, nursing managers and nursing educators should implement intervention measures to improve self-efficacy and promote feedback-seeking behavior, so as to reduce the adverse impact of transition shock on nursing interns and reduce the loss of clinical nursing staff.

## Data Availability

The raw data supporting the conclusions of this article will be made available by the authors, without undue reservation.

## Footnotes

^1^ www.wjx.com
